# Differentially Expressed Genes Identify FIGO Stage II Cervical Cancer Patients with a Higher Risk of Relapse in a Small Cohort

**DOI:** 10.3390/jpm15100497

**Published:** 2025-10-16

**Authors:** Carolina P. S. Melo, Angelo B. Melo, Fábio R. Queiroz, Álvaro P. Costa, Laurence R. Amaral, Ramon A. Pereira, Izabela F. G. Amorim, Jorge G. G. Ferreira, Wander J. Jeremias, Pedro L. L. Bertarini, Matheus S. Gomes, Letícia C. Braga, Paulo G. O. Salles

**Affiliations:** 1 Laboratory of Translational Research in Oncology, Teaching, Research and Innovation Center, Mario Penna Institute, Belo Horizonte 30380-490, MG, Brazil; carolina.melo@mariopenna.org.br (C.P.S.M.); fabio.queiroz@mariopenna.org.br (F.R.Q.); ramon.pereira@mariopenna.org.br (R.A.P.); izabela.amorim@professor.faminas.edu.br (I.F.G.A.); jorge.ferreira@mariopenna.org.br (J.G.G.F.); 2 Laboratory of Bioinformatics and Molecular Analysis, Federal University of Uberlandia, Patos de Minas 38702-178, MG, Brazil; angelo.melo@ufu.br (A.B.M.); laurence@ufu.br (L.R.A.); bertarini@ufu.br (P.L.L.B.); matheusgomes@ufu.br (M.S.G.); 3 Postgraduate Program in Sciences Applied to Surgery and Ophthalmology, Faculty of Medicine, Federal University of Minas Gerais, Belo Horizonte 30130-100, MG, Brazil; alvaro.costa@mariopenna.org.br; 4 Laboratory of Experimental Pharmacology, School of Pharmacy, Federal University of Ouro Preto, Ouro Preto 35402-163, MG, Brazil; wander.jeremias@ufop.edu.br

**Keywords:** cervical cancer prognosis, gene expression signature, FIGO staging, machine learning model, cervical cancer recurrence, artificial intelligence for oncology, network-based selection method

## Abstract

**Background/Objectives**: Most studies investigating prognostic biomarkers in cervical cancer (CC) analyze patients irrespective of FIGO stage, potentially masking molecular features that underlie the aggressiveness of some FIGO II tumors. To address this, we investigated differential gene expression in a FIGO II CC cohort to identify a gene signature predictive of progression-free survival (PFS) within five years of treatment initiation. **Methods**: Tumor samples from 15 CC patients were analyzed using RNA sequencing, bioinformatics, and machine learning to identify differentially expressed genes (DEGs) associated with prognosis. Findings were validated in an independent CC cohort (*n* = 174). **Results**: High expression of B3GALT1 (HR = 5.11), GTF3C2-AS1 (HR = 18.73), and ZKSCAN4 (HR = 5.18) was significantly associated with an increased risk of recurrence in our cohort. Elevated expression of these transcripts is also associated with shorter PFS in the external dataset. Notably, GTF3C2-AS1 expression alone was sufficient to classify all fifteen patients into their respective prognostic groups using a decision tree model, achieving 93.3% accuracy in leave-one-out cross-validation (LOOCV). Additional candidates, including RCAN2-DT, MYH9-DT, IGKC, IGHG1, and IGHG3, were associated with PFS in our cohort but could not be externally validated due to a lack of available data. **Conclusions**: Transcriptomic profiling revealed potential biomarkers that refine prognostic stratification in cervical cancer beyond FIGO staging. Among them, GTF3C2-AS1 consistently emerged as a potential predictor of recurrence risk. Additional candidates, including B3GALT1, ZKSCAN4, and immunoglobulin transcripts, provided complementary insights but require further validation. These preliminary results highlight intra-stage heterogeneity in FIGO II CC and underscore the promise of molecular markers to improve risk assessment.

## 1. Introduction

Cervical cancer (CC) ranks as one of the most lethal types of cancer, precisely the fourth leading cause of death among females globally. Significant investments have been made in CC diagnosis and treatment, including the development of vaccines, highlighting economic disparities among countries. In 2022, about 94% of CC-related deaths occurred in less developed countries [1].

Diagnoses have increased with the expansion of CC screening programs, underscoring the urgent need to enhance disease stratification strategies. The International Federation of Gynecology and Obstetrics (FIGO) staging system is the predominant method used worldwide for evaluating prognosis in CC patients. Its latest update in 2018 incorporated imaging techniques and pathological features, such as lymph node involvement and tumor size, to improve prognostic outcomes’ differentiation and effectively guide clinical management [2]. A key advantage of the updated FIGO staging system is its flexibility in utilizing clinical, radiological, or pathological findings to determine stage assignment, an approach more inclusive, especially when considering the limited resources of less developed countries [3,4,5,6].

FIGO stage II in CC is characterized by the carcinoma invasion beyond the uterus into the surrounding tissues, but without extension to the lower third of the vagina or the pelvic wall [7]. It is subdivided into IIA and IIB, depending on the region where the carcinoma has spread. Although with a relatively good prognosis compared to stages III and IV, the estimated 5-year survival rate is around 66% for IIB patients [2], exposing an intragroup heterogeneity. Considering the inherent complexity of tumors, CC molecular features may be responsible for the observed differences in outcomes, and their identification could improve treatment stratification and clinical management for CC patients.

The concept of using molecular markers for patient stratification in CC is not new. With the increasing prevalence of studies employing next-generation sequencing (NGS), there has been substantial progress in understanding the influence of molecular features on aspects ranging from disease susceptibility to progression and survival, facilitating the identification of potential biomarkers and therapeutic targets [8]. In recent years, multiple prognostic biomarkers for CC have been described; however, their clinical application remains limited due to insufficient validation in large cohorts or difficulty in implementation. Su et al. reported an association between Zac1 expression and poor prognosis in CC [9]. Zhao et al. developed and validated an expression-based prognostic signature comprising SPP1, EFNA1, MMP1, ITM2A, and DSG2 genes as an independent predictor of CC survival [10]. Similarly, Ju et al. identified a five-mRNA (GALNTL6, ARSE, DPAGT1, GANAB, and FURIN) prognostic signature related to post-translational modifications for predicting both disease-free and overall survival in CC [11].

The studies describing CC prognostic biomarkers often group patients regardless of their FIGO stage. One of the few exceptions is the study of Nguyen et al., who proposed a 70-gene signature for predicting treatment outcomes comparing early-stage (stage I–IIA) and advanced-stage (stage IIB–IV) CC [12]. Although often grouped as advanced-stage disease, FIGO stage III and IV CC are characterized by more extensive disease, with an expected poorer prognosis, whereas FIGO stage II is more heterogeneous. Stage IIB in particular is considered locally advanced; however, some patients may still experience prolonged progression-free survival [13]. Thus, a biomarker able to identify, at diagnosis, FIGO II patients who will present shorter progression-free survival (PFS) after treatment could be used as an additional tool in the management of high-risk patients.

To address this hypothesis, this study investigated the differential gene expression in a FIGO II CC cohort between patients with good and poor prognoses. We first profiled the differentially expressed genes (DEGs) of FIGO II CC patients from our cohort, based on which genes were up- or downregulated in patients with recurrent and non-recurrent disease after treatment. Subsequently, we evaluated the impact of these genes on the hazard ratio (HR) for PFS. Furthermore, a machine learning (ML) analysis was performed using normalized gene expression counts to identify potential biomarker candidates for improved stratification of FIGO II CC patients with distinct prognoses. The prognostic value of these candidate biomarkers was further validated using data from The Cancer Genome Atlas (TCGA) cohort. Our findings provide novel insights into the molecular heterogeneity underlying FIGO stage II CC and may support clinicians in identifying high-risk patients warranting closer clinical surveillance.

## 2. Materials and Methods

### 2.1. Patient Recruitment and Sample Selection

The study was approved by the Institutional Ethics Committee (CAAE: 41114915.5.0000.5121). Tumor samples from fifteen patients with CC were collected at the Mario Penna Institute (Belo Horizonte, Brazil) from August 2017 until May 2019. Patients who met the inclusion criteria, such as no previous history of cancer or any immune diseases, histopathological diagnosis of adenocarcinoma or squamous cell carcinoma (SCC), diagnosis of cervical cancer at stage II according to the FIGO staging system, and who had signed the informed consent form, were included. After the diagnosis and biopsy, the patients underwent chemoradiotherapy, and clinical data were collected and analyzed. Patients were classified as having either a poor prognosis (PP) or a good prognosis (GP), based on PFS being lower than or higher than five years, respectively.

### 2.2. General View of the Study

**Figure 1:**
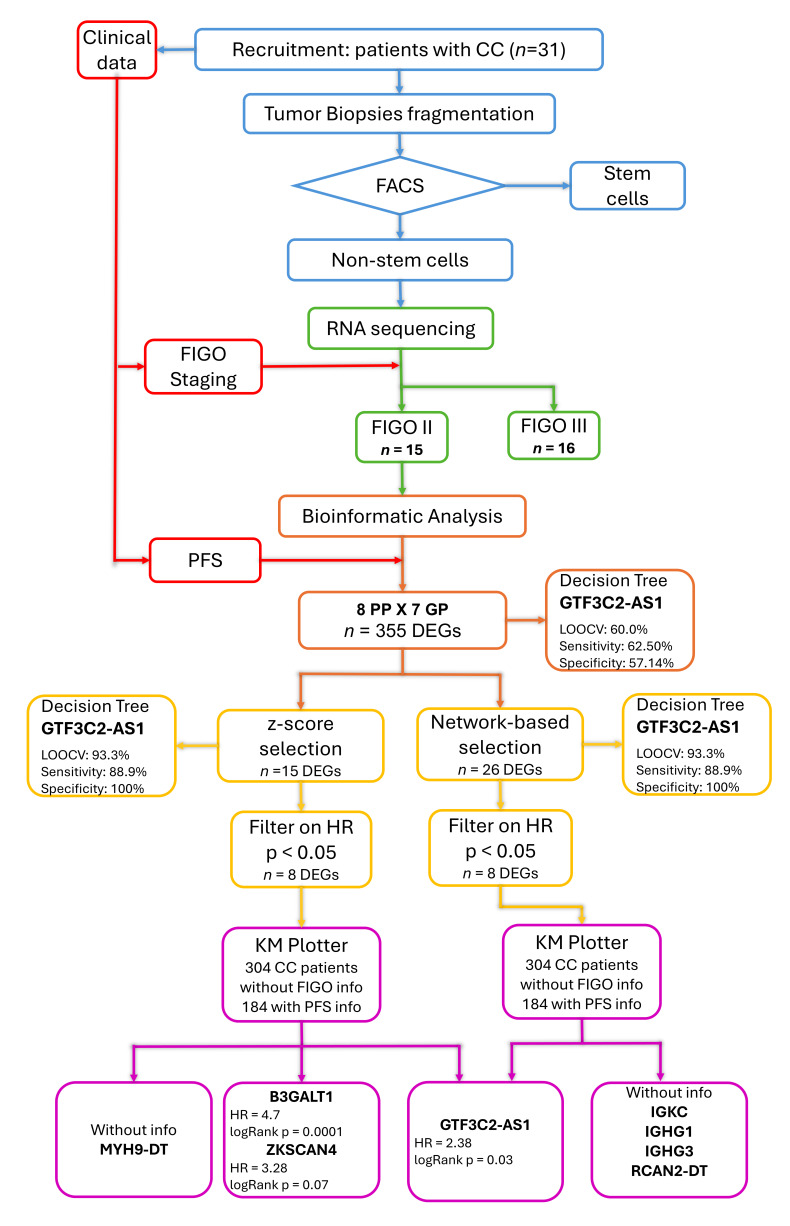
Schematic representation of the study workflow. The colors indicate the different methodological steps: cervical cancer non-stem cell selection (blue), clinical data collection (red), transcriptome sequencing (green), identification of differentially expressed genes (DEGs) between poor (PP) and good (GP) prognosis groups (orange), variable selection considering their association with progression-free survival (PFS) measured as hazard ratio (HR) (yellow), and validation in an external cohort (purple).

This work is part of a previous study of our group, which investigated DEGs in CC stem-like cells (CCSCs) from tumor biopsies at diagnosis between patient responders and non-responders to chemoradiotherapy [14]. In the present study only the non-stem cells fraction was used. The flow diagram of this study is presented in Figure 1.

### 2.3. Fluorescence-Activated Cell Sorting (FACS)

The protocol for cell sorting has been previously described [14]. Briefly, FACS was used to isolate enriched CCSCs from a complex mixture of tumor cells based on their light scatter and fluorescent staining profiles. CC tissue fragments (5 mm) from patients’ biopsies were disaggregated using the BD™ Medimachine System according to the manufacturer’s instructions (BD-Biosciences, Franklin Lakes, NJ, USA). The resulting cell suspension was frozen in 20% HES cryoprotective solution (100 mL anhydrous glucose 1.7 g/L; Na (+1) 140 mEq/L; Cl (−1) 98 mEq/L; K (+1) 5 mEq/L; Mg (+2) 3 mEq/L; Gluconate 23 mEq/L; Acetate 27 mEq/L) and stored in liquid nitrogen until use. Two monoclonal antibodies corresponding to the cell surface markers CD45 (APC-H7 Clone 2D1) and CD34 (PE Clone 563) were used in the cell suspension, increasing the cell concentration to 5 × 10^6^ cells/mL. CCSCs-enriched subpopulations were isolated using the FACSAria^®^ flow-sorter (BD-Biosciences, Franklin Lakes, NJ, USA). Yield mode was performed at 45 psi with 85-μm nozzle at a frequency of ~51 kHz. CCSCs were sorted into cytometry tubes using a gate containing the CD45−/CD34+ population to eliminate contamination with hematopoietic stem cells. Non-stem cells were kept for subsequent RNA sequencing.

### 2.4. Next Generation Sequencing (NGS)

The non-stem cell pellet was processed with the SMART-Seq v4 Ultra-low Input RNA Sequencing Kit (Takara Bio USA, Mountain View, CA, USA) for cDNA synthesis, according to the manufacturer’s instructions. Qualitative and quantitative analysis of cDNA were performed using the Agilent High Sensitivity DNA Chip run on the Agilent Bioanalyzer 2100 (Agilent, Santa Clara, CA, USA) and the Qubit™ dsDNA HS Assay Kit on a Qubit 3 Fluorometer (Thermo Fisher Scientific, Waltham, MA, USA), respectively. cDNA suitable for NGS presented fragments from 400 bp to 10,000 bp, and concentration ≥ 0.3 ng/μL.

Sequencing libraries were prepared using the Nextera XT Library Prep Kit (Illumina, San Diego, CA, USA) and the Nextera XT Index Kit V2 Set A (Illumina, San Diego, CA, USA), following the manufacturer’s instructions. The libraries’ fragment size was measured using the High Sensitivity D1000 kit on the 2200 TapeStation System (Agilent, Santa Clara, CA, USA). Libraries’ concentration was determined with the Qubit™ dsDNA HS Assay Kit on a Qubit 3 Fluorometer (Thermo Fisher Scientific, Waltham, MA, USA). NGS was performed using a NextSeq^®^ 500/550 High Output Kit v2 (150 cycles) on a NextSeq^®^ 550 sequencer (Illumina, San Diego, CA, USA).

### 2.5. Differential Gene Expression Analysis

Raw reads obtained from RNA-Seq underwent quality assessment using FastQC (version 0.11.9) [15]. Adapters and sequences with lengths or Phred scores lower than 35 and 30, respectively, were discarded by Trimmomatic (version 0.39) [16]. Filtered reads were aligned to the Homo Sapiens reference genome, version GRCh38, using the STAR software (version 2.7.10b) [17] and the GTF file from the same genome obtained from Ensembl (version 109) [18]. The gene expression quantification was performed using the “outFilterMultimapNmax 1” and “quantMode GeneCounts” STAR options. The raw counts were filtered using HTSFilter (version 1.48) [19] and then normalized with DESeq2 (version 1.48.1) [20]. Both packages are part of the R software environment (version 4.3.2) [21]. Finally, differential gene expression analysis was performed using DESeq2 (version 1.38.3). Only DEGs with log2FoldChange (log2FC) > 1 or <−1 and *p*.adjusted (*p*adj) value < 0.05 were considered. After removing duplicated genes, hierarchical clustering analysis was performed to group upregulated and downregulated DEGs, using the pheatmap R package (version 1.0.13) [22].

Two approaches were used to select the transcripts most associated with patients PFS. In the first method the initial DEGs list were filtered to include those that were upregulated (z-score > 0) in at least 50% of the PP group and, simultaneously, downregulated (z-score < 0) in at least 50% of the GP group, or vice versa. The z-score values were calculated with the following equation:

(1) z = (x − Mean(x))/SD(x),

where

- x = data frame with normalized counts obtained from DESeq2;
- Mean(x) = mean expression of each DEG across all patients, calculated using the R basic function *rowMeans*;
- SD(x) = standard deviation across all patients, calculated using the *rowSds* function from the matrixStats R package (version 1.5) [23].

The second approach employed a network-based variable selection strategy using the regnet R package (version 1.0.2) [24]. This method not only incorporates correlations among genomic features but also applies a network-based penalization under the accelerated failure time (AFT) model, combined with Kaplan–Meier weights. By accounting for correlations in survival outcomes, this approach has been widely applied for variable selection in high-dimensional cancer genomic datasets [24]. Gene expression, as normalized counts, and PFS time were used as input data. The response and penalty parameters were set to “survival” and “network”, respectively, and the robust method was chosen. To compute the optimal value of lambda, the number of folds for cross-validation was set to 15.

### 2.6. Statistical Analysis

Five-year progression-free and overall survival (OS) rates were assessed through Kaplan–Meier method and compared through the Cox–Mantel (log-rank) test using the Kaplan–Meier (KM) plotter web-based platform [25,26]. HR’s considering PFS time were obtained through Cox regression using the ‘coxph’ function from the ‘survival’ R package (version 4.4.1) [27] for each variable selected. Patients were divided into high and low groups based on gene expression levels above or below the median. High expression levels were set to 1, and low expression levels were set to 0. Log-rank test *p*-values < 0.05 were considered significant.

Machine learning (ML) analysis using normalized counts from the DEGs was performed via the J48 algorithm in WEKA software (Waikato Environment for Knowledge Analysis, version 3.6.11, University of Waikato, Hamilton, New Zealand) [28]. Briefly, this algorithm builds decision trees based on a set of training data, selecting at each node the attribute that most effectively splits the samples into subsets. To reduce the likelihood of overfitting, the Confidence Factor (C) parameter, used for pruning the decision tree, was set to 0.25, and the Minimum Number of Instances per Leaf (M) parameter, which prevents the creation of nodes based on very few samples, was set to 2. A higher value for this parameter typically results in smaller, more generalizable trees. No hyperparameter optimization was performed outside the validation loop, ensuring that all model adjustments occurred exclusively within the cross-validation process to avoid information leakage.

Leave-one-out cross-validation (LOOCV) was applied to evaluate test performance, which is particularly suitable for small datasets. In this approach, each of the 15 samples was systematically left out once, with the model trained on the remaining 14 samples, and the procedure repeated until every sample had served as the test instance exactly once. The classification accuracy calculated represents the average performance across all folds, reflecting the model’s overall generalizability rather than the performance of a single model.

### 2.7. Validation with an External Cohort

Searches were conducted for larger CC expression datasets across different databases; however, only one dataset included PFS information in the clinical data, while the other two provided only OS (Table 1).

For this reason, the prognostic value of the selected genes was evaluated in an independent cohort using the Kaplan–Meier (KM) plotter web-based platform [25,26]. This tool enables real-time survival analysis based on transcriptomic data from large, publicly available patient cohorts. A cervical squamous cell carcinoma cohort comprising 304 patients was selected under the “Pan Cancer RNA-seq” option. Since FIGO stage information was not available for this dataset, all patients with documented recurrence status were included. Samples were stratified into two groups (high and low expression) using the median expression values for each one of the selected genes that presented significant HR values (*p* < 0.05). Survival analyses were performed using recurrence-free survival (RFS) as the clinical endpoint. The follow-up threshold was set at 60 months, and patients were censored beyond this time point. Group comparisons were assessed using the Cox–Mantel (log-rank) test. Differences in gene expression between high and low groups were observed using boxplot and compared through Mann–Whitney test in GraphPad Prism 9.0 (GraphPad Software, Boston, MA, USA).

## 3. Results

In this study, we aimed to identify a gene signature differentially expressed in FIGO II CC patients who are more likely to experience relapse within five years of starting treatment. Tumor biopsies from 15 women with a median age of 48 years (range 24–81) were submitted to RNA sequencing of their non-stem cell fraction. The cohort clinicopathological characteristics are summarized in Table 2 (for details, please see Table S1). Approximately 93.3% of patients (14/15) had SCC, with only one patient having adenocarcinoma. Stage FIGO IIB was predominant (14/15), and one patient was classified as FIGO IIA. The mean tumor size was 6.1 cm, and most patients had parametrial (93.3%) and/or vaginal involvement (93.3%). Most tumors were moderately (53%) or poorly differentiated (40%), and one was undifferentiated (grade IV).

**Figure 2:**
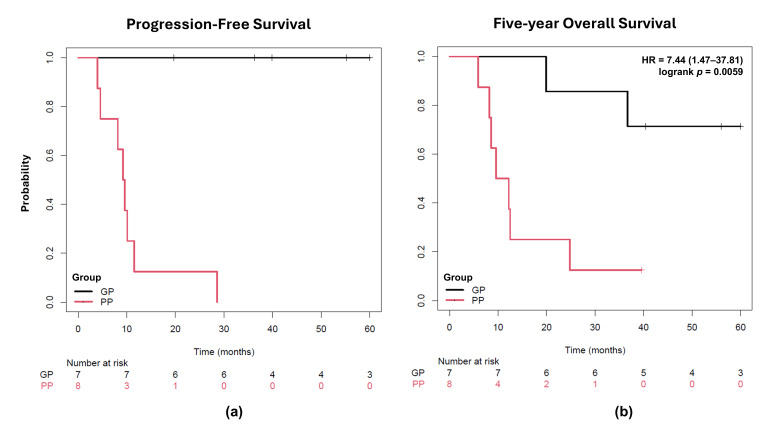
Kaplan–Meier plots for cervical cancer patients after 5 years of follow-up. (**a**) Progression-free survival was considered for a period of five years after diagnosis. (**b**) Overall survival in five years was compared using the Cox–Mantel (log-rank) test. The occurrence of metastasis after treatment was used as a criterion for patient assignment in the PP group and was significantly associated with shorter 5-yr overall survival. PP: poor prognosis; GP: good prognosis; HR: hazard ratio.

Patients were categorized into two prognostic groups based on whether they presented or did not present distant metastasis after treatment. The PP group was characterized by a lower median age at diagnosis (39 years, ranging from 24 to 67), tumor size greater than 4 cm (88%), and unilateral parametrial involvement in most cases (76%). On the other hand, the GP group was characterized by a higher median age at diagnosis (64 years, ranging from 32 to 82) and bilateral parametrial involvement in most cases (71%). Patients classified as PP had a median PFS of 9.4 months, with 5-yr OS rate of only 12.5% (HR = 7.44, 95% CI 1.47–37.81, *p* < 0.01) (Figure 2). In contrast, GP patients exhibited a PFS of more than 60 months and a 5-year OS rate of 74.2%.

**Figure 3:**
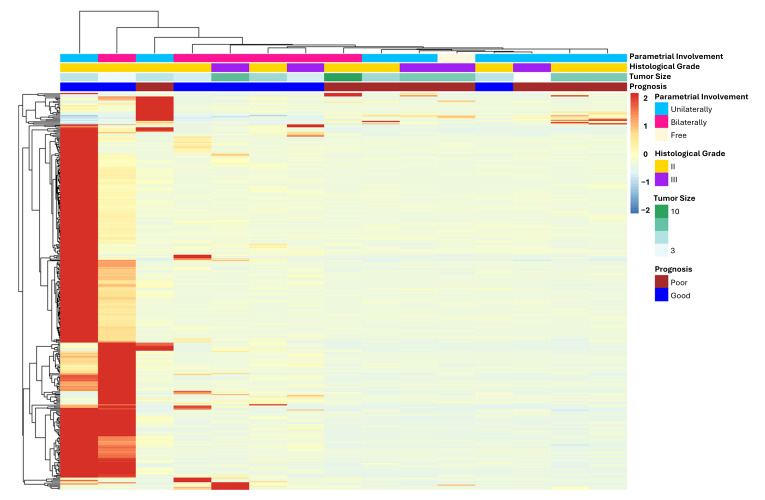
Heatmap of differentially expressed genes between FIGO II CC patients with poor and good prognosis. Only DEGs with log2FC > 1 or <−1 and padj value < 0.05 were considered, totaling 355 genes. Most of them are upregulated in only two GP patients (left side of the heatmap).

The comparison between gene expression data of both groups revealed 355 DEGs associated with patient outcome (Tables S2 and S3), most of them were upregulated in only two GP patients (Figure 3).

**Figure 4:**
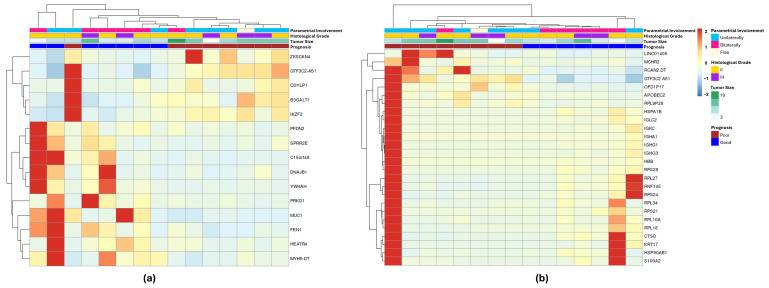
Differentially expressed genes associated with FIGO II CC patients’ prognosis selected by two different approaches. (**a**) Heatmap for the 15 DEGs selected by the z-score method: upregulated in at least 50% of the PP group and, at the same time, downregulated in at least 50% of the GP group, or vice versa. (**b**) Heatmap for the 26 DEGs selected by network-based method. GTF3C2-AS1 was the only transcript in common between the two lists.

To identify the transcripts most strongly associated with patient prognosis, two approaches were applied. In the first analysis, we filtered the dataset for genes that were upregulated in at least 50% of the PP group and simultaneously downregulated in at least 50% of the GP group, or vice versa, based on z-score values (Table S4). Fifteen transcripts were selected based on these criteria (Tables S5–S7), and the resulting heatmap is depicted in Figure 4a. The second analysis used a network-based variable selection strategy, which incorporates correlations among genomic features and accounts for correlations in survival outcomes. Twenty-six transcripts were selected through this method (Tables S8 and S9) and are shown as a heatmap in Figure 4b. Only the GTF3C2-AS1 transcript was selected by the two methods.

Given the small sample size of our cohort, we sought larger CC gene expression datasets in public repositories to validate the identified prognostic signature. Among the three available RNA-seq datasets, only one included PFS information. Therefore, we utilized the KMplotter web-based platform to assess the relationship between gene expression and PFS in an independent CC cohort. This dataset comprised RNA-seq profiles from 304 CC samples, of which 184 had available PFS data. As FIGO stage information was not provided, analyses could not be restricted to stage II patients.

**Figure 5:**
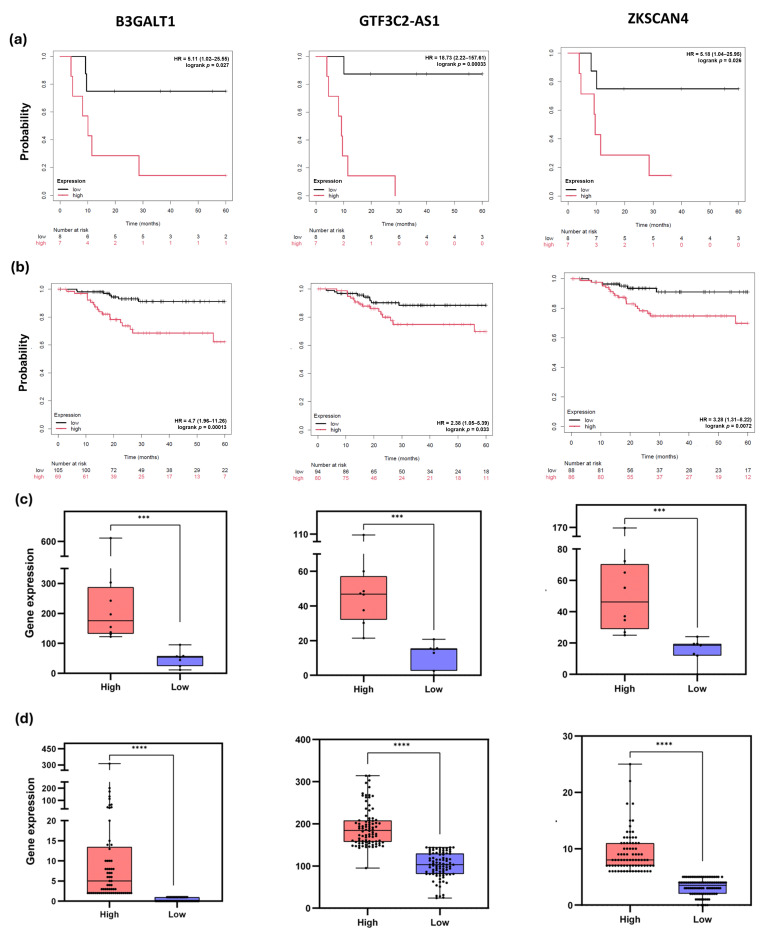
DEGs significantly associated with PFS in the internal and external cohorts. (**a**,**b**) Kaplan–Meier plots of PFS for the (**a**) internal (*n* = 15) and (**b**) the external (*n* = 174) cohorts. Curves were generated by KMplotter platform using the expression values for the genes associated with PFS in both cohorts. The results suggest that high expression of B3GALT1, GTF3C2-AS1, or ZKSCAN4 significantly increases the risk of disease recurrence within five years. (**c**,**d**) Boxplot of patients’ distribution into high (red) and low (blue) groups based on gene expression level above or below the median. Gene expressions were significantly different between both groups for the three DEGs, either in (**c**) the internal or (**d**) the external cohort. The box extends from the 25th to the 75th percentiles. The line in the middle of the box is plotted at the median. Whiskers were plotted from the minimum to the maximum value. Each dot corresponds to one different patient. Comparison was performed using the Mann–Whitney test. *** *p* < 0.001; **** *p* < 0.0001.

Univariable Cox regression identified eight (60%) of the 15 DEGs from the z-score selection method as significantly associated with PFS (Table S10), three of which (B3GALT1, GTF3C2-AS1, ZKSCAN4) were validated in an external CC cohort (*n* = 174) (Table 3). Higher expression of these transcripts correlated with increased relapse risk and shorter PFS (Figure 5). MYH9-DT showed a protective effect in our cohort (HR = 0.1, 95% CI: 0.01–0.85, *p* = 0.011), though external validation was not possible due to lack of data. Other transcripts (IKZF2, MUC1, PRKD1, YWHAH) were significant internally but not externally.

From the network-based selection method, eight of 26 transcripts were associated with PFS internally (Table S10), but only GTF3C2-AS1 (also identified by the z-score method) was validated externally (Table 4). Four transcripts (IGHG1, IGHG3, IGKC, RCAN2-DT) were unavailable in KMplotter; in our dataset, immunoglobulin genes suggested a protective role (HR < 1), while RCAN2-DT correlated with poor prognosis.

Decision tree analysis reinforced GTF3C2-AS1 as a key discriminator between patient groups. While full training achieved perfect classification using all 355 DEGs, leave-one-out cross-validation (LOOCV) resulted in modest performance, with 60% accuracy, 62.5% sensitivity (correctly classifying PP patients), and 57.1% specificity (correctly classifying GP patients). In contrast, restricting the input to transcripts selected by the z-score or network-based methods markedly improved model performance, achieving 93.3% accuracy in LOOCV, with 88.9% sensitivity and 100% specificity. These parameters are summarized in Table 5. These results highlight the importance of dimensionality reduction for enhancing predictive power.

## 4. Discussion

Cervical cancer stands out as the leading cause of cancer-related deaths among women, with approximately 660,000 new cases in 2022, according to the World Health Organization. The FIGO staging system is crucial for classifying patient risk and guiding treatment decisions. However, it still does not fully capture the complexity of the molecular tumor’s response to chemotherapy and its inherent heterogeneity. In this context, molecular tests could provide additional information to staging systems, thereby refining the segregation of patients into risk groups. For instance, the updated FIGO 2023 staging for endometrial cancer incorporates, among other parameters, molecular classification, influencing stages I and II [31]. In this study, we identified three DEGs with the potential to subclassify the FIGO II stage CC into good and poor prognosis. Patients with higher expression of B3GALT1, GTF3C2-AS1, and ZKSCAN4 presented an increased risk of disease recurrence in our cohort. Interestingly, the same pattern was observed in CC patients with shorter PFS time using a larger external dataset.

The B3GALT1 is a member of the beta-1,3-galactosyltransferase (beta3GalT) gene family. Its role in cancer is controversial. High levels of B3GALT1 have been shown to enhance CD8+ T cell infiltration and reduce immune evasion, suppressing breast cancer lung metastasis [32]. On the other hand, higher expression of B3GALT1 results in higher levels of MUC1-associated Sialyl Lewis antigen, which is linked to the metastasis of prostate cancer cells [33]. In this study, we observed higher levels of B3GALT1 in CC patients who present metastasis after treatment, corroborating the association observed in prostate cancer. ZKSCAN4, also referred to as ZNF307, belongs to the Krüppel-associated box zinc finger (KRAB-ZFPs) family, which is the largest group of zinc finger transcription factors [34]. Researchers have shown that ZKSCAN4 reduces cell proliferation, migration, and invasion in a hepatocellular carcinoma model. Coherently, the knockdown of ZKSCAN4 restored the proliferative phenotype [35]. Interestingly, our study revealed that ZKSCAN4 was upregulated in CC patients with poor prognosis. This finding contrasts with its role in hepatocellular carcinoma, suggesting a context-specific function of ZKSCAN4 in different cancer types. GTF3C2-AS1 codes for a lncRNA for which there is limited information regarding its mechanism of action. Nonetheless, it has been associated with poor OS in endometrial carcinoma [36], corroborating our findings. In our study, PP patients exhibited higher expression levels of GTF3C2-AS1. Moreover, a decision tree model successfully classified all patients based solely on GTF3C2-AS1 expression, achieving 93.33% accuracy in predicting prognosis. This result underscores the potential of GTF3C2-AS1 as a promising candidate for a prognostic biomarker in CC.

Gene expression data for five of the transcripts identified as associated with PFS in our cohort were not available in the KMplotter CC patient database. Two of them are divergent transcripts, MYH9-DT and RCAN2-DT. According to HGNC, divergent transcripts are a subgroup of long noncoding RNA (lncRNA) located within 300–500 nucleotides of a protein-coding gene on the opposite strand. It has been suggested that these lncRNA positively regulate the transcription of nearby genes [37]. Thus, we could assume that the upregulation of MYH9-DT would lead to increased levels of MYH9 transcripts. The MYH9 gene encodes non-muscle myosin II-A (NMM-IIA), a protein essential for various cellular functions, including cell division, adhesion, and migration. Its role in cancer is controversial, being implicated as an oncogene or a tumor suppressor, depending on the specific cancer type [38]. One study has shown that using direct RNA interference (RNAi) to silence Myh9 promotes the formation of invasive SCC in tumor-susceptible models, likely due to NMM-IIA’s role in regulating post-transcriptional p53 stabilization [39]. In this context, the association between the elevated expression of MYH9-DT and patients’ good prognosis observed here could be a consequence of higher p53 stabilization resulting from increased MYH9 transcript levels, ultimately improving their response to chemotherapy. In the same way, the upregulation of the RCAN2-DT would lead to increased levels of RCAN2 transcripts. RCAN2 is one of the regulators of the calcineurin protein family and participates in phosphorylation by inhibiting the activation of calcineurin. Its role in cancer is still unclear. It was described as involved in tumor progression in gastric cancer [40], whereas its overexpression has been demonstrated to enhance apoptosis in vitro and in vivo [41]. In our cohort, RCAN2-DT higher levels were associated with shorter PFS, in consonance with the findings in gastric cancer.

The other three transcripts absent from the validation dataset were immunoglobulins significantly associated with better prognosis in our cohort. IGKC (immunoglobulin kappa constant) has previously been linked to metastasis-free survival in breast cancer and identified as a prognostic marker in non-small-cell lung cancer and colorectal cancer [42]. More recently, IGKC expression was associated with favorable response to immune checkpoint blockade in melanoma [43]. IGHG1 and IGHG3, which encode the immunoglobulin heavy constant gamma 1 and 3 chains, respectively, have been reported with heterogeneous prognostic roles. Several studies have demonstrated that high IGHG1 expression promotes tumor proliferation, migration, and invasion, and correlates with poor prognosis [44,45,46]. Conversely, other reports describe IGHG1 and IGHG3 as suppressor genes in breast cancer recurrence [47]. In addition, elevated IGHG3 expression was observed in advanced non-small-cell lung cancer patients who responded to carboplatin plus paclitaxel chemotherapy compared with non-responders [48]. The strong association of IgG with cellular immunity, together with the high correlation of IGHG transcripts with metagenes of adaptive immune cells, suggests that elevated expression of these genes may reflect an active antitumor immune response, which may be responsible for the protective association observed in our patients [49].

Despite current limitations, the application of artificial intelligence (AI) to predict CC outcomes shows considerable promise for improving patient care by supporting clinical decision-making through the integration of genomic and clinical data [50]. In our study, decision tree analysis using the complete set of 355 DEGs was less accurate than models built after variable reduction. Decision trees based on selected transcripts achieved markedly higher sensitivity and specificity, underscoring the importance of rigorous variable selection in ML-based approaches. Using the z-score method, we identified transcripts that most effectively distinguished the two prognostic groups; 60% (8/15) were significantly associated with PFS in our cohort, and three (20%) were also validated in an external cohort. By contrast, the network-based method identified a lower proportion of transcripts that were significantly associated with PFS internally, only 30% (8/26). External validation of these findings was limited, as four of the eight transcripts—three of them immunoglobulin genes—were not available in the external dataset. Given the established role of immunoglobulin transcripts in tumor metastasis and prognosis, it is likely that their relevance will also extend to other CC cohorts, although further validation is required. Importantly, the two selection methods provided complementary insights into the molecular mechanisms underlying CC recurrence. Notably, GTF3C2-AS1 was the only transcript identified by both methods and was also prioritized by the decision tree analyses, where its expression alone was sufficient to stratify patients into the correct risk groups.

It is important to emphasize that this is an exploratory study and the findings should be considered preliminary. A key limitation was the absence of a large independent CC cohort that matched our specific criteria—patients with FIGO stage II disease and PFS data from a 5-year follow-up. Given the small size of our cohort, such validation would have been particularly valuable. As a feasible alternative, we used the KMplotter platform CC dataset to assess associations between gene expression and PFS in an external cohort, although FIGO stage information was unavailable for these patients. Despite these limitations, our results are intriguing and warrant further validation. Moreover, this study addresses a relatively underexplored question: the heterogeneity within patients classified under the same clinical stage.

## 5. Conclusions

This study highlights the potential of transcriptomic profiling to refine prognostic stratification in cervical cancer (CC) beyond the current FIGO staging system. We identified three transcripts—B3GALT1, ZKSCAN4, and GTF3C2-AS1—consistently associated with poor prognosis in FIGO stage II CC, with GTF3C2-AS1 emerging as a potential biomarker capable of classifying patients by risk with high accuracy. Additional candidates, including divergent transcripts (MYH9-DT, RCAN2-DT) and immunoglobulin genes (IGKC, IGHG1, IGHG3), revealed prognostic associations in our cohort, further suggesting their involvement in CC outcomes, although external validation remains limited.

Importantly, this exploratory study underscores the biological heterogeneity within patients assigned to the same FIGO stage, raising the value of molecular markers for more precise risk classification. Despite the limitations imposed by cohort size and incomplete external datasets, our findings provide preliminary but intriguing evidence that transcript-based signatures, particularly GTF3C2-AS1, may serve as clinically relevant prognostic biomarkers in CC. Further validation in larger, well-annotated cohorts are warranted.

## Figures and Tables

**Table 1:** Public datasets with larger CC gene expression acquired through RNA sequencing.

	TCGA-CESC	CGCI-HTMCP-CC	KMplotter
Method	RNAseq	RNAseq	RNAseq
Cases	306	212	304
Cases with Gene Expression	303	123	304
FIGO II Stage cases	70	91	NA
Cases with Treatment Outcome	39	34	174
OS time	Yes	Yes	Yes
PFS time	No	No	Yes
Reference	[29]	[30]	[26]

TCGA: The Cancer Genome Atlas; CESC: Cervical Squamous Cell Carcinoma and Endocervical Adenocarcinoma Collection; CGCI: Cancer Genome Characterization Initiative; HTMCP: HIV+ Tumor Molecular Characterization Project; CC: cervical cancer; KM: Kaplan Meier; RNA: ribonucleic acid; FIGO: International Federation of Gynecology and Obstetrics; NA: not available; OS: overall survival; PFS: progression free survival.

**Table 2:** Clinicopathological characteristics of cervical cancer patients.

	PP Group *n* = 8	GP Group *n* = 7	Total (%) *n* = 15
Median age (years)	39	64	48
Diagnosis			
SCC	7 (88%)	7 (100%)	14 (93%)
Adenocarcinoma	1 (12%)	-	1 (7%)
Histological grade			
II	4 (50%)	4 (57%)	8 (53%)
III	3 (38%)	3 (43%)	6 (40%)
IV	1 (12%)	-	1 (7%)
FIGO stage			
IIA	1 (12%)	-	1 (7%)
IIB	7 (88%)	7 (100%)	14 (93%)
Tumor size (cm)			
>4 cm	7 (88%)	4 (57%)	11 (73%)
≤4 cm	-	3 (43%)	3 (20%)
NA	1 (12%)	-	1 (7%)
Parametrial Involvement			
Bilaterally	1 (12%)	5 (71%)	6 (40%)
Unilaterally	6 (76%)	2 (29%)	8 (53%)
Free	1 (12%)	-	1 (7%)
Vaginal Involvement			
Present	8 (100%)	4 (57%)	12 (80%)
Absent	-	2 (29%)	2 (13%)
NA	-	1 (14%)	1 (7%)
Distant metastasis after treatment			
Yes	8 (100%)	-	8 (53%)
No	-	7 (100%)	7 (47%)

SCC: Squamous Cell Carcinoma. PP: poor prognosis; GP: good prognosis; FIGO: International Federation of Gynecology and Obstetrics; NA: not available.

**Table 3:** Hazard ratios for transcripts selected by z-score method significantly associated with PFS in the internal cohort.

	Progression-Free Survival HR (95% CI), *p*	
mRNA	Internal Cohort (*n* = 15)	External Cohort (*n* = 174)
B3GALT1	5.11 (1.02–25.55) ***p*** **= 0.027**	4.7 (1.96–11.26) ***p*** **= 0.0001**
GTF3C2-AS1	18.73 (2.22–157.61) ***p*** **= 0.0003**	2.38 (1.05–5.39) ***p*** **= 0.033**
IKZF2	6.74 (1.33–34.24) ***p*** **= 0.0087**	1.24 (0.56–2.74) *p* = 0.59
MUC1	0.07 (0.01–0.58) ***p*** **= 0.002**	1.84 (0.81–4.17) *p* = 0.14
MYH9-DT	0.1 (0.01–0.85) ***p*** **= 0.011**	NA
PRKD1	0.09 (0.01–0.74) ***p*** **= 0.0052**	2.14 (0.94–4.85) *p* = 0.063
YWHAH	0 (0–inf) ***p*** **= 0.0001**	0.85 (0.39–1.86) *p* = 0.68
ZKSCAN4	5.18 (1.04–25.95) ***p*** **= 0.026**	3.28 (1.31–8.22) ***p*** **= 0.0072**

*p*-values in bold were considered significant (*p* < 0.05). HR: hazard ratio; CI: confidence interval; mRNA: messenger ribonucleic acid; B3GALT1: Beta-1,3-Galactosyltransferase 1; GTF3C2-AS1: General Transcription Factor IIIC Subunit 2 Antisense RNA 1; IKZF2: IKAROS Family Zinc Finger 2; MUC1: Mucin 1; MYH9-DT: Myosin Heavy Chain 9 Divergent Transcript; PRKD1: Protein Kinase D1; YWHAH: Tyrosine 3-Monooxygenase/Tryptophan 5-Monooxygenase Activation Protein Eta; ZKSCAN4: Zinc Finger with KRAB and SCAN Domains 4; NA: not available.

**Table 4:** Hazard ratios for transcripts selected by the network-based method significantly associated with PFS in the internal cohort.

	Progression-Free Survival HR (95% CI), *p*	
mRNA	Internal Cohort (*n* = 15)	External Cohort (*n* = 174)
GTF3C2_AS1	18.73 (2.22–157.61) ***p*** **= 0.0003**	2.38 (1.05–5.39) ***p*** **= 0.033**
HSPA1B	0.22 (0.04–1.13) ***p*** **= 0.049**	1.23 (0.56–2.72) *p* = 0.6
IGHG1	0.07 (0.01–0.58) ***p*** **= 0.002**	NA
IGHG3	0.07 (0.01–0.58) ***p*** **= 0.002**	NA
IGKC	0.19 (0.04–0.99) ***p*** **= 0.03**	NA
KRT17	0.07 (0.01–0.58) ***p*** **= 0.002**	1.31 (0.59–2.89) *p* = 0.5
RCAN2-DT	5.64 (1.12–28.49) ***p*** **= 0.019**	NA
RNF145	0.19 (0.04–0.99) ***p*** **= 0.03**	1.01 (0.46–2.22) *p* = 0.98

*p*-values in bold were considered significant (*p* < 0.05). HR: hazard ratio; CI: confidence interval; mRNA: messenger ribonucleic acid; GTF3C2-AS1: General Transcription Factor IIIC Subunit 2 Antisense RNA 1; HSPA1B: Heat Shock Protein Family A (Hsp70) Member 1B; IGHG1: Immunoglobulin Heavy Constant Gamma 1; IGHG3: Immunoglobulin Heavy Constant Gamma 3; IGKC: Immunoglobulin Kappa Constant; KRT17: Keratin 17; RCAN2-DT: Regulator Regulator of Calcineurin 2 Divergent Transcript; RNF145: Calcineuri Ring Finger Protein 145; NA: not available.

**Table 5:** Decision tree analyses performance parameters in LOOCV.

	Input Data		
Parameter	All DEGs (*n* = 355)	z-Score Selection (*n* = 15)	Network-Based Selection (*n* = 26)
Accuracy	60.0%	93.3%	93.3%
Specificity	57.1%	100%	100%
Sensitivity	62.5%	88.9%	88.9%

DEGs: Differentially Expressed Genes.

## Data Availability

The data presented in this study are openly available in SRA database, reference number PRJNA812529.

## Supplementary Materials

The following supporting information can be downloaded at: https://www.mdpi.com/article/10.3390/jpm15100497/s1, Table S1: Clinical Data; Table S2: DESeq2 Results 355 DEGs; Table S3: 355 DEGs; Table S4: zscore 355 DEGs; Table S5: DESeq2 results 15 DEGs; Table S6: 15 DEGs; Table S7: zscore 15 DEGs; Table S8: 26_DEGs; Table S9: zscore_26_DEGs; Table S10: Hazard_Ratios_for_selected_DEGs.

## Abbreviations

The following abbreviations are used in this manuscript:

CAAE	Certificate of Presentation of Ethical Appreciation
CC	Cervical Cancer
DEGs	Differentially Expressed Genes
FACS	Fluorescence-activated cell sorting
FIGO	The International Federation of Gynecology and Obstetrics
GP	Good Prognosis
HR	Hazard Ratio
lncRNA	Long noncoding RNA
log2FC	log2 Fold Change
LOOCV	Leave-One-Out Cross-Validation
NGS	Next Generation Sequencing
OS	Overall Survival
padj	p adjusted
PP	Poor Prognosis
PFS	Progression-free Survival
SCC	Squamous Cell Carcinoma
TCGA	The Cancer Genome Atlas
